# Performance of Nucleic Acid Amplification Tests for Detection of Severe Acute Respiratory Syndrome Coronavirus 2 in Prospectively Pooled Specimens

**DOI:** 10.3201/eid2701.203379

**Published:** 2021-01

**Authors:** Hannah Wang, Catherine A. Hogan, Jacob A. Miller, Malaya K. Sahoo, ChunHong Huang, Kenji O. Mfuh, Mamdouh Sibai, James Zehnder, Brendan Hickey, Nasa Sinnott-Armstrong, Benjamin A. Pinsky

**Affiliations:** Stanford University School of Medicine, Stanford, California, USA (H. Wang, C.A. Hogan, J.A. Miller, M.K. Sahoo, C. Huang, J. Zehnder, N. Sinnott-Armstrong, B.A. Pinsky);; Stanford Health Care, Stanford (K.O. Mfuh, M. Sibai, B.A. Pinsky);; Stanford University, Stanford (N. Sinnott-Armstrong)

**Keywords:** coronaviruses, virus, severe acute respiratory syndrome coronavirus 2, SARS-CoV-2, coronavirus disease, COVID-19, testing, diagnostic screening programs, performance, nucleic acid amplification tests, prospectively pooled specimens, respiratory infections, zoonoses

## Abstract

Pooled nucleic acid amplification tests for severe acute respiratory syndrome coronavirus 2 could increase availability of testing at decreased cost. However, the effect of dilution on analytical sensitivity through sample pooling has not been well characterized. We tested 1,648 prospectively pooled specimens by using 3 nucleic acid amplification tests for severe acute respiratory syndrome coronavirus 2: a laboratory-developed real-time reverse transcription PCR targeting the envelope gene, and 2 commercially available Panther System assays targeting open reading frame 1ab. Positive percent agreement (PPA) of pooled versus individual testing ranged from 71.7% to 82.6% for pools of 8 and from 82.9% to 100.0% for pools of 4. We developed and validated an independent stochastic simulation model to estimate effects of dilution on PPA and efficiency of a 2-stage pooled real-time reverse transcription PCR testing algorithm. PPA was dependent on the proportion of tests with positive results, cycle threshold distribution, and assay limit of detection.

The ability of clinical laboratories to meet the demand for severe acute respiratory syndrome coronavirus 2 (SARS-CoV-2) testing is critical for reducing coronavirus disease (COVID-19)–related illness, death, and economic impact. Pooled testing has the potential to decrease resources required for population-level screening and can provide valuable data to inform public health policies (*1*,*2*). Several previous experimental and modeling studies have demonstrated the feasibility of pooled SARS-CoV-2 nucleic acid amplification testing (NAAT), in pools of <32 individual samples (*3*–*9*). However, the potential increase in efficiency gained by pooled testing is offset by a theoretical dilution-related decrease in analytical sensitivity (*8*,*10*).

Despite this decrease in sensitivity, pooled testing of blood donors for transfusion-transmitted infections, such as those with HIV-1 and hepatitis C virus, has proven to be safe and effective (*11*). This efficacy varies depending on the performance characteristics of the assay, the prevalence of infection, viral load kinetics, and pooling size, and strategy. For agents with variable seasonal or geographic prevalence, such as West Nile virus, many blood banks use adaptive risk-based pooling strategies, switching from pooled to individual testing when there is an increase in regional prevalence (*12*). Adapting a similar risk-based pooling strategy for SARS-CoV-2 has the potential to enable more widespread testing of high-risk populations and asymptomatic critical infrastructure workers, guide aggressive contact-tracing measures, and help direct public health interventions to where they are most needed. However, there are limited prospective data on assay-specific performance characteristics of pooled testing to guide implementation of such a strategy. Furthermore, there is little evidence on parallel test performance of different assays on pooled samples to direct choice of method.

In this study, we aimed to evaluate the test performance characteristics of 1 laboratory-developed and 2 commercially available SARS-CoV-2 NAATs for 1,648 individual respiratory specimens prospectively grouped in pools of 8 and 4. We used these data to validate a stochastic model to estimate optimal pool size, efficiency, and expected positive percent agreement (PPA) of a 2-stage pooled testing algorithm that takes into account prevalence, viral load distribution, and assay analytical sensitivity.

## Methods

### Clinical Specimens

The Stanford Clinical Virology Laboratory receives samples from tertiary-care academic hospitals and affiliated outpatient facilities in the San Francisco Bay Area of California. Prospective pooling of consecutive nasopharyngeal or oropharyngeal swab specimens submitted for SARS-CoV-2 testing during the morning shift was conducted during June 10–19, 2020, for evaluation of a pool size of 8 and during July 6–July 23, 2020, for evaluation of a pool size of 4. Samples submitted for testing were collected from symptomatic and asymptomatic inpatients and outpatients, either for clinical care or in the context of COVID-related epidemiologic surveillance studies and drug trials at our institution. As samples from persons enrolled in these studies and trials were received daily in batches, they were randomly evenly distributed among pools on a daily basis. This distribution was conducted to preserve the independence between samples in the same pool; these samples had not been tested before receipt in our laboratory and were otherwise treated identically to nonresearch samples. Nonresearch samples were otherwise assigned to pools consecutively. Additional laboratorywide data on proportion of tests positive and cycle threshold (C_t_) value distribution were obtained from all specimens (n = 74,162) tested during March 1–June 24, 2020. This study was conducted with Stanford institutional review board approval (protocol no. 48973), and individual consent was waived.

### Pool Size Determination

In this study, an initial pool size of 8 was selected on the basis of pilot experiments with pool sizes ranging from 4 to 10 (B.A. Pinsky, unpub. data), and the logistical consideration that pooling in multiples of 4 would be more efficient for the robotic liquid handlers in our laboratory. After review of the test performance characteristics of 8-sample pooling in conjunction with the results of an independent stochastic simulation model, additional testing was performed to evaluate a pool size of 4 to generate empiric data for further model validation. Subset analyses of first tests versus follow-up tests were conducted by retrospectively assigning pools to 1 of the 2 groups on the basis of the status of the positive sample(s) in that pool. Pools containing positive samples belonging to both groups were excluded from this analysis. To validate the performance of the model for additional pool sizes, an external in silico dataset was obtained on the basis of pool sizes of 3 and 5. The in silico analysis was performed according to US Food and Drug Administration recommendations (Appendix) (*13*).

### Sample Pooling, Extraction, and NAAT

Pools were constructed before nucleic acid extraction by combining 500 μL from each of the individual samples. For a pool size of 8, this resulted in a total volume of 4 mL and a dilution factor of 1:8. For a pool size of 4, this resulted in a total volume of 2 mL and a dilution factor of 1:4.

Subsequently, total nucleic acids were extracted from 500 μL taken from each pool and each individual specimen by using QIAsymphony and the QIAsymphony DSP Virus/Pathogen Midi Kit (QIAGEN, https://www.qiagen.com) and eluted into 60 μL of AVE buffer according to manufacturer’s instructions. Real-time reverse transcription PCR (rRT-PCR) was performed by using an emergency use authorization laboratory-developed test (LDT) targeting the envelope gene with the Rotor-Gene Q Instrument (QIAGEN) as described (*14*–*16*), with pooled samples tested on the same run as component individual samples. A C_t_ result of 40–45 was considered an indeterminate result, which was adjudicated by repeat testing and resulted as positive if reproducible with an acceptable amplification curve. Specimens were only reported as negative if the internal control human RNase P gene was detected at a C_t_<35.

On the same day as QIAsymphony extraction, another 500 μL from each pool was transferred to a Hologic Panther Specimen Lysis Tube (Hologic, https://www.hologic.com) and tested by using the Panther Fusion SARS-CoV-2 Assay (Hologic) and Panther Aptima SARS-CoV-2 assay (Hologic) per the manufacturer’s recommendations (*17*,*18*). In addition to the manufacturer-set cutoff value, receiver operating characteristic (ROC) curve analysis of pooled relative light unit (RLU) values, with individual test results as the reference method, was used to determine the optimal RLU discrimination threshold. A focused electronic medical record review was conducted for all samples.

### Statistical Analysis

ROC curve analysis was conducted by using R package pROC (*19*). PPA and negative percent agreement (NPA) were calculated by using individual testing as the reference method and were reported with exact (Clopper-Pearson) 95% CIs (*20*). Passing-Bablok regression was used to compare C_t_ values of the individual LDT, pooled LDTs, and pooled Panther Fusion assays. The 95% CIs of slope, intercept, and bias were calculated by using an ordinary nonparametric bootstrap resampling method with default parameters in R package mcr. Paired *t*-tests were used to compare the mean differences between paired C_t_ values among different assays. A Student *t*-test was used to compare the mean difference between internal control RNase P C_t_ values in false-negative and true-negative pools. All comparisons were 2-sided with type I error set at 0.05. We used the laboratory-wide C_t_ value distribution and a separate limit of detection (LoD) experiment to develop a stochastic simulation model to estimate PPA and efficiency for a 2-stage pooled testing algorithm, which was subsequently validated by using the independent empiric pools of 8 and pools of 4 data, as well as in silico pools of 5 and pools of 3 data. We provide the methods used to develop this model (Appendix).

## Results

### Assay Comparisons for Pools of 8

**Figure 1:**
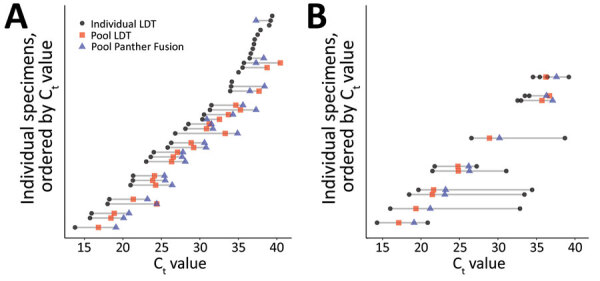
Performance of nucleic acid amplification tests for detection of severe acute respiratory syndrome coronavirus 2 in prospectively pooled specimens. For a pool size of 8, paired individual and pooled C_t_ values for each individually positive sample (n = 58), in order of increasing individual C_t_ value. A) Pools comprising only 1 positive sample/pool. B) Pools comprising >2 positive samples/pool. The gray lines span the range of C_t_ values associated with a given pool. Rows without gray lines indicate individually positive samples belonging to pools that were negative by both real-time reverse transcription PCR methods. Panther Fusion is from Hologic (https://www.hologic.com). C_t_, cycle threshold; LDT, laboratory-developed test.

To evaluate a pool size of 8, a total of 112 pools from 896 samples were each tested on 3 different NAAT platforms (Table 1). Two pools were invalid, 1 by the Panther Fusion assay (0.9%), and 1 by the Panther Aptima assay (0.9%), and were excluded from subsequent analysis. All 16 individual samples in these 2 pools showed negative results. The remaining 110 pools contained 880 individual samples. Four samples were tested in duplicate in 2 different pools and showed identical results. Among the 880 individual samples, 58 (6.6%) showed positive results and a median C_t_ value of 31.4 (interquartile range 22.1–35.5). First-time diagnostic specimens had a higher median C_t_ value than specimens that underwent follow-up tests (Table 2). ROC curve analysis for the Panther Aptima showed optimal cutoff values between 343 and 393 RLUs; a cutoff value of 350 was chosen as the nearest round number (Panther Aptima-350) (Appendix Table 1, Figure 1).

**Table 1:** Performance of nucleic acid amplification tests for detection of severe acute respiratory syndrome coronavirus 2 in prospectively pooled specimens, by testing platform*

Test name	Gene target(s)	Internal control	Method	Strategy	Reference
LDT	Envelope	RNase P	rRT-PCR	Pools of 8†, pools of 4†	(*1*,*14*–*16*)
Panther Fusion	ORF1ab	Reagent spike-in	rRT-PCR	Pools of 8†, pools of 5†, pools of 3‡	(*17*)
Panther Aptima-M	ORF1ab	Reagent spike-in	TMA	Pools of 8 with manufacturer-set RLU cutoff†	(*18*)
Panther Aptima-350	ORF1ab	Reagent spike-in	TMA	Pools of 8 with RLU cutoff of >350†§	(*18*)

*Panther Aptima-M, Panther Aptima with manufacturer-set relative light unit cutoff value; Panther Aptima-350, Panther Aptima with relative light unit cutoff value >350 considered positive. Both products were from Hologic (https://www.hologic.com). LDT, laboratory-developed test; ORF1ab, open reading frame 1ab; rRT-PCR, real-time reverse transcription PCR; RLU, relative light unit; TMA, transcription-mediated amplification.

†Pooled testing strategy was assessed empirically at Stanford Clinical Virology Laboratory, with individual samples evaluated by LDT.

‡Pooled testing strategy assessed by in silico sensitivity analysis, with individual samples evaluated by Panther Fusion.

§Panther Aptima RLU cutoff of 350 selected based on receiver operating characteristic curve (Appendix Figure 1).

**Table 2:** Proportion of tests positive for severe acute respiratory syndrome coronavirus 2 with median C_t_ values in pooled testing and laboratorywide clinical testing datasets, subset by testing indication*

Dataset	No. positive samples/no. total samples (%)				Median C_t_ value (IQR)		
	All	First	Follow-up		All	First	Follow-up
Pools of 8†	58/880 (6.6)	24/657 (3.7)	34/223 (15.2)		31.4 (22.1–35.5)	24.4 (18.4–33.1)	34.1 (29.0–36.8)
Pools of 4‡	38/768 (4.9)	28/491 (5.7)	10/277 (3.6)		29.3 (20.3–33.9)	27.5 (19.4–32.6)	32.2 (24.9–34.5)
Hologic§	10,000/52,272 (19.1)	NA	NA		26.2 (20.7–32.6)	NA	NA
Laboratory-wide¶	1,358/74,162 (1.8)	1,109/66,070 (1.7)	249/8,092 (3.1)		28.5 (23.0–34.3)	27.2 (22.2–32.4)	34.2 (29.0–37.4)
March	555/8,896 (6.2)	489/8,557 (5.7)	66/339 (19.5)		26.7 (21.9–31.5)	26.4 (21.8–31.2)	28.6 (22.6–35.2)
April	518/22,671 (2.3)	404/21,167 (1.9)	114/1,504 (7.5)		30.6 (24.8–36.0)	28.8 (22.7–34.6)	35.4 (32.9–38.0)
May	172/21,833 (0.8)	136/19,505 (0.7)	36/2,328 (1.5)		27.5 (23.3–34.7)	26.1 (22.5–31.3)	35.4 (30.4–37.3)
June	113/20,762 (0.5)	80/16,841 (0.5)	33/3,921 (0.84)		28.2 (21.2–33.6)	27.4 (21.3–32.7)	30.6 (20.2–34.4)

*Ct, cycle threshold; IQR, interquartile range; NA, not available.

†Pools of 8 specimens were tested in our clinical laboratory during June 10–19, 2020.

‡Pools of 4 specimens were tested in our clinical laboratory during July 6–23, 2020.

§Hologic dataset comprises specimens tested clinically by Panther Fusion (https://www.hologic.com) during March 1–July 31, 2020 at 2 different external sites. These data were used to perform in silico sensitivity analysis to evaluate pool sizes of 3 and 5.

¶Composed of clinical specimens obtained during March 1–June 24, 2020.

Among the tested pools of 8, a total of 41.8% (46/110) contained >1 positive sample. The positive pools comprised 36 pools with 1 positive sample, 9 pools with 2 positive samples, and 1 pool with 4 positive samples (Table 3). There were 3 false-positive pools, 1 on each platform, in which each of the individual samples showed negative results. The overall PPA of pooled testing ranged from 71.7% to 82.6%, and NPA ranged from 98.4% to 100.0% (Table 4). The 14 pools containing positive first-time diagnostic samples had higher PPAs than the 28 pools containing positive follow-up test samples in an LDT (Appendix Table 3).

**Table 3:** Results of 8-sample pooled testing, by testing platform and number of positive specimens per pool (n = 110) for detection of severe acute respiratory syndrome coronavirus 2*

Pool no.	Pooled testing				Individual testing		Total no. pools
LDT	Panther Fusion	Panther Aptima-M	Panther Aptima-350	Positive, (no. 1 PP, no. >1 PP)	Negative
1	+	+	+	+	30 (21, 9)	0	30
2	+	+	–	+	2 (1, 1)	0	2
3	+	+	–	–	0 (0, 0)	0	0
4	+	–	+	+	0 (0, 0)	0	0
5	+	–	–	+	0 (0, 0)	0	0
6	+	–	–	–	1 (1, 0)	1†	2
7	–	+	+	+	2 (2, 0)	0	2
8	–	+	–	+	1 (1, 0)	0	1
9	–	+	–	–	0 (0, 0)	1‡	1
10	–	–	+	+	2 (2, 0)	0	2
11	–	–	–	+	1 (1, 0)	1§	2
12	–	–	–	–	7 (7, 0)	61	68
No. positive pools	34	36	34	39	46 (36, 10)	–	–
No. negative pools	76	74	76	71	–	64	–
Total no. pools	110	110	110	110	–	–	110

*Panther Aptima with manufacturer-set relative light unit cutoff. Panther Aptima-350, Panther Aptima with relative light unit cutoff value >350 was considered positive; Ct, cycle threshold; LDT, laboratory-developed test; Pos, positive; RLU, relative light unit; 1 PP, 1 positive specimen in pool; >1 PP, >2 positive specimens in pool; –, negative; +, positive.

†False-positive LDT Ct value was 37.5.

‡False-positive Panther Fusion Ct value was 38.8.

§False-positive Panther Aptima-350 RLU value was 434.

**Table 4:** Performance characteristics and efficiency of 8-sample and 4-sample pooled testing, by testing platform (n = 302), for detection of severe acute respiratory syndrome coronavirus 2 in prospectively pooled specimens*

Testing platform	Pool size	PPA, % (95% CI)	NPA, % (95% CI)	Pools positive, %	Average test run/sample
LDT	8	71.7 (56.5–84.0)	98.4 (91.5–100.0)	30.9	0.434
Panther Fusion	8	76.1 (61.2–87.4)	98.4 (91.5–100.0)	32.7	0.452
Panther Aptima-M	8	73.9 (58.9–85.7)	100.0 (94.3–100.0)	30.9	0.434
Panther Aptima-350	8	82.6 (68.6–92.2)	98.4 (91.5–100.0)	34.5	0.470
LDT	4	94.3 (80.8–99.3)†	100 (97.7–100.0)	17.2	0.422
Panther Fusion‡	4	100.0 (85.8–100.0)	100 (96.7–100.0)	17.6	0.426
Panther Aptima-M	4	82.9 (66.2–93.4)†	100 (97.7–100.0)	15.1	0.401
Panther Aptima-350	4	88.6 (73.3–96.8)†	100 (97.7–100.0)	16.2	0.411

*Panther Aptima-M, Panther Aptima with manufacturer-set RLU cutoff value. Panther Aptima-350, Panther Aptima with RLU cutoff value >350 was considered positive. Both products from Hologic (https://www.hologic.com). LDT, laboratory-developed test; NPA, negative percent agreement; PPA, positive percent agreement; RLU, relative light unit.

†Restricting the performance characteristics comparison to only the 136 pools tested by Panther Fusion resulted in a PPA as follows: LDT 100% (95% CI 85.8%–100.0%), Aptima-M 91.7% (95% CI 73.0%–99.0%), and Aptima-350 95.8% (95% CI 78.9%–99.9%).

‡A total of 56 of the 192 pools tested on the other platforms were not tested by Panther Fusion.

There were 16 total pools for which >1 method showed false-negative results. Except for the 1 pool containing 4 positive specimens, which was not detected by Panther Aptima using the manufacturer’s cutoff value (Panther Aptima-M), the remaining 15 false-negative pools each contained only 1 positive specimen. For all missed pools, the C_t_ value of the individual positive sample was >34 (median 36.6, interquartile range 35.5–37.7) (Figure 1). Among individual positive specimens in the dataset for pools of 8, a total of 22 (37.9%) had C_t_ values >34. A total of 13/22 (59.1%) were false negative for the LDT, 11/22 (50.0%) for the LDT Panther Fusion, 15/22 (68%) for the LDT Panther Aptima-M, and 8/22 (36.4%)for the LDT Panther Aptima-350. Each of these false-negative samples was collected from known symptomatic or convalescent-phase patients being monitored for viral clearance; none of these samples were initial diagnostic specimens. The pooled LDT RNase P internal control C_t_ values were similar in false-negative (mean 23.5, 95% CI 22.7–24.3) and true-negative (mean 23.4, 95% CI 22.7–24.1; p = 0.7) pools.

### Linearity Studies for Pools of 8

**Figure 2:**
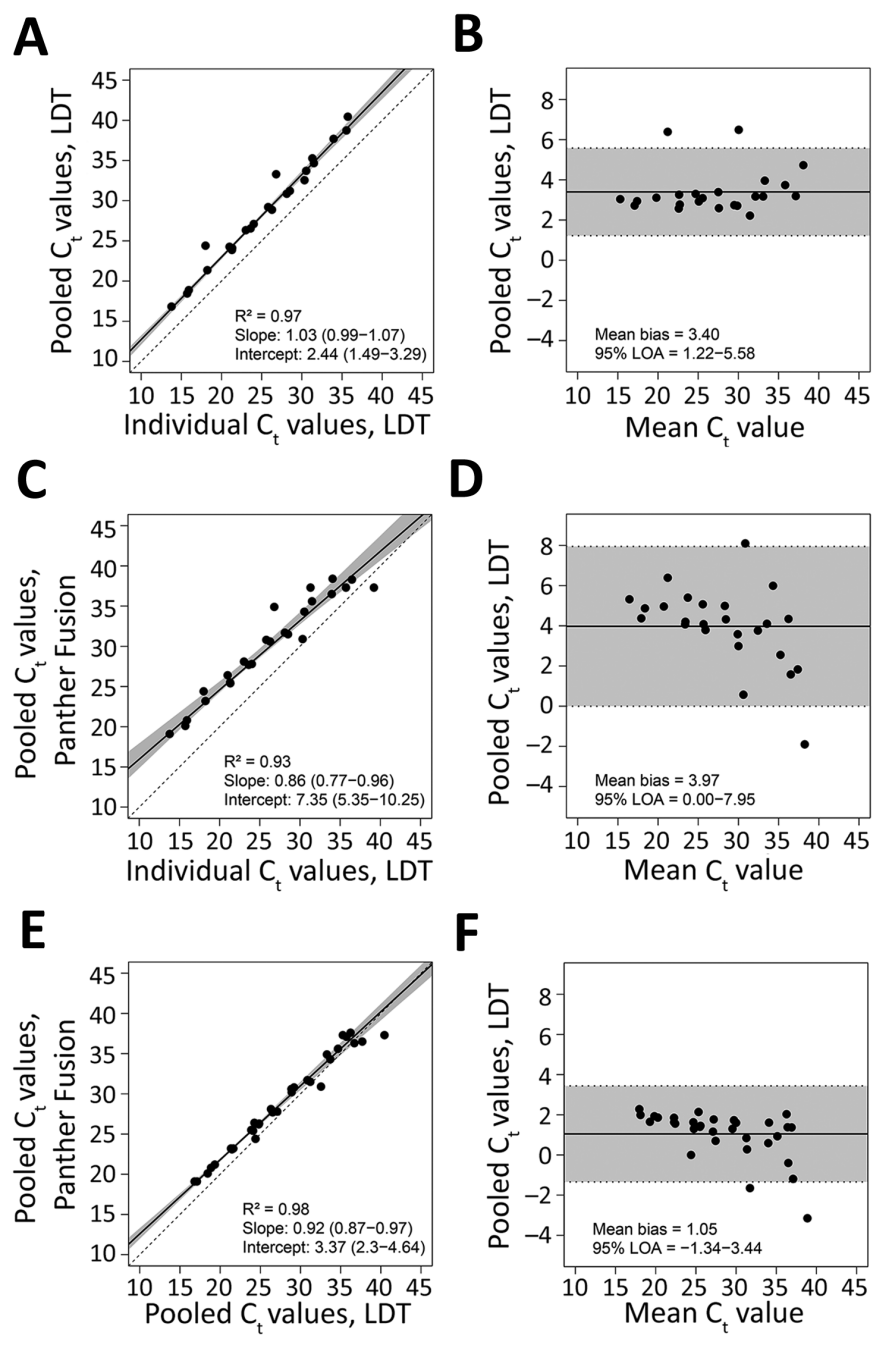
Performance of nucleic acid amplification tests for detection of severe acute respiratory syndrome coronavirus 2 in prospectively pooled specimens. Passing-Bablok regression and Bland-Altman plots for pools of 8 containing only 1 positive sample, tested by A and B) pooled LDT versus individual LDT (n = 23) (A, B); pooled Panther Fusion versus individual LDT (n = 25) (C, D); and pooled Panther Fusion versus pooled LDT (n = 32) (E, F). For the Passing-Bablok regression plots (A, C, and E), the solid line indicates the line of regression. 95% CIs are shaded in gray. The dashed line indicates the line of identity. The slope and intercept of the regression line are reported with 95% CIs in parentheses. For the Bland-Altman plots (B, D, and F), the solid line represents the mean difference in C_t_ value. 95% limits of agreement are shaded in gray. Panther Fusion is from Hologic (https://www.hologic.com). C_t_, cycle threshold; LDT, laboratory-developed test; LOA, limits of agreement.

For pools containing only 1 positive sample, the pooled rRT-PCRs showed positive systematic bias when compared with the individual LDT assay, as shown by the Passing-Bablok regression intercept value being >0. Mean bias between pooled and individual C_t_ values was 3.4 cycles (95% limits of agreement 1.2–5.6; p<0.001) by LDT and 4.0 cycles (95% limits of agreement 0.0–8.0; p<0.001) by Panther Fusion (Figure 2). Panther Fusion showed negative proportional bias when compared with individual and pooled LDTs, as shown by Passing-Bablok regression slopes with 95% CIs that do not contain 1. This result is additionally highlighted in the Bland-Altman plots, which demonstrate that at higher C_t_ values, Panther Fusion outperforms the LDT.

### Model Estimates

The modeled PPA estimate is sensitive to the input parameters of proportion of positive tests, assay analytical sensitivity, and viral load distribution. The analytical sensitivity of the assay is approximated in this model by the C_t_ value corresponding to the probability of detecting 95% of true-positive samples, otherwise known as the 95% LoD. Specimens with C_t_ beyond the LoD are assigned a decreasing probability of detection on the basis of a probit regression curve, the shape of which was determined in the initial validation of the LDT (Appendix Figure 5). The viral load distribution of the tested population is approximated in this model by the proportion of samples with C_t_ greater than the LoD. This makes the model output independent of the actual LoD C_t_ value itself, enabling the model to be used across different rRT-PCRs.

**Figure 3:**
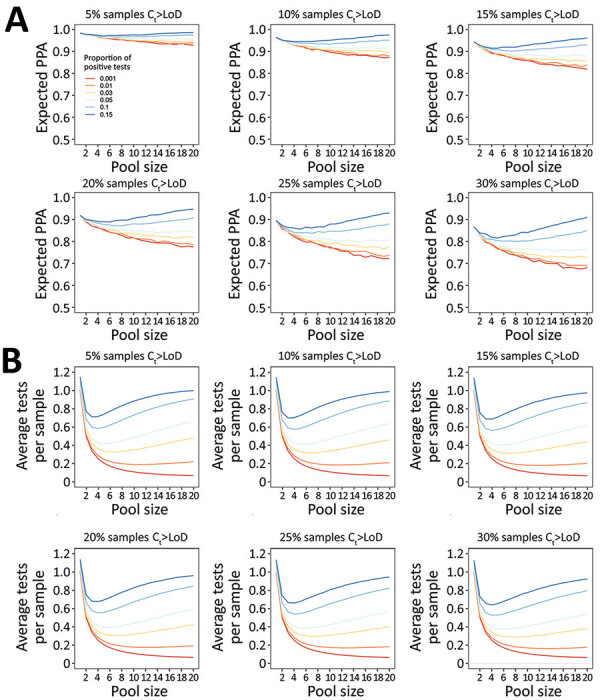
Performance of nucleic acid amplification tests for detection of severe acute respiratory syndrome coronavirus 2 in prospectively pooled specimens. Model-estimated PPA and testing efficiency, by pool size, proportion of tests positive, and proportion of samples with C_t_ above the 95% LoD. For these estimates, LoD has been held constant at the experimentally-derived C_t_ of 35.9, although results are independent of specific LoD value. A) Expected PPA between pooled and individual testing at pool sizes of 1–20. PPA decreases with decreasing proportion of test results positive (indicated by colored lines in each plot), and with increasing proportion of samples with C_t_ values beyond the 95% LoD (each panel). At >5% test positivity, expected PPA starts to increase at larger pool sizes because there is a greater likelihood of 2 positive samples being in the same pool. The baseline PPA (pool size of 1) reflects the likelihood of obtaining the same individual result with repeat (nonpooled) testing. B) Estimated average tests per sample that would be performed at each pool size, with a lower number of average tests per sample corresponding to higher testing efficiency. Efficiency increases with decreasing proportion of test results positive, and slightly increases with increasing samples with C_t_ above the LoD. Each missed pool results in fewer deconvolutions, and thus fewer total tests performed. C_t_, cycle threshold; LoD, limit of detection; PPA, positive percent agreement.

**Figure 4:**
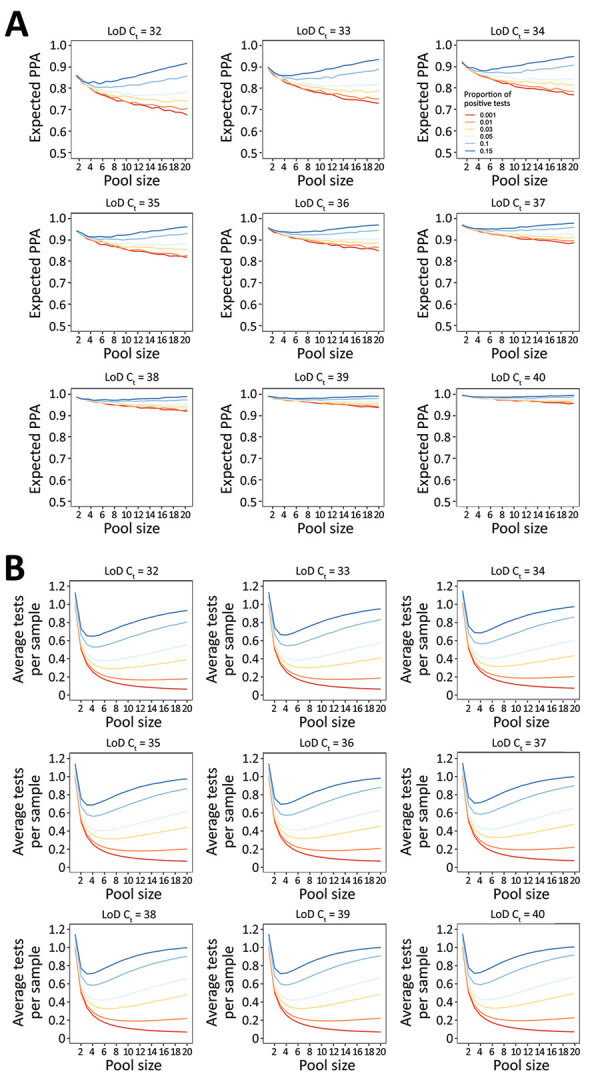
Performance of nucleic acid amplification tests for detection of severe acute respiratory syndrome coronavirus 2 in prospectively pooled specimens. Model-estimated PPA and testing efficiency, by pool size, proportion of tests positive, and assay analytical sensitivity as approximated by the C_t_ corresponding to the 95% LoD. For these estimates, the population viral load distribution has been held constant at 15% of samples with C_t_ values >35. A) Expected PPA between pooled and individual testing at pool sizes of 1–20. PPA decreases with decreasing proportion of tests positive (indicated by colored lines in each plot) and increases with increased analytical sensitivity (each panel). This result occurs because the proportion of individual samples with a C_t_ value above each LoD decreases as the C_t_ LoD increases. B) Estimated average tests per sample that would be performed at each pool size, with a lower number of average tests per sample corresponding to higher testing efficiency. Efficiency increases with decreasing proportion of test results positive, and slightly decreases with increased analytical sensitivity because more pools detected results in an increased number of individual tests performed at the deconvolution step. C_t_, cycle threshold; LoD, limit of detection; PPA, positive percent agreement.

If the assay analytical sensitivity is kept constant, but the tested population changes such that a greater proportion have a C_t_ value beyond the 95% LoD, PPA decreases (Figure 3, panel A). Conversely, if the patient population is kept constant, but assay analytical sensitivity increases (i.e., from lower C_t_ LoD to higher C_t_ LoD), PPA increases (Figure 4, panel A). However, if assay analytical sensitivity changes and the tested population shifts accordingly such that it retains the same proportion C_t_ >LoD, then the PPA stays constant (Appendix Figure 6). In contrast, the average expected tests per sample is almost entirely determined by pool size and prevalence, whereas analytical sensitivity (LoD C_t_) and the underlying C_t_ distribution minimally affect efficiency because of small absolute numbers of false-positive pools (Figure 3, panel B; Figure 4, panel B). To achieve a 5% absolute difference in efficiency with an increase in LoD C_t_ from 32 to 40, a prevalence of 25% would be required.

Both PPA and tests per sample are highly dependent on pool size and prevalence of infection. As prevalence increases, PPA can counterintuitively increase with larger pool sizes because there is a greater likelihood of having more than 1 positive sample in a given pool, which would be expected to increase PPA. Similarly, test efficiency can decrease with larger pool sizes because the likelihood of deconvoluting a positive pool increases. Estimated PPA and average tests per sample for inputs of percentage of positive tests 0.1%–15.0% and proportion of samples with Ct value above the LoD ranging from 5% to 30% are available (Appendix Table 4).

### Model Sensitivity Analyses and Validation

**Figure 5:**
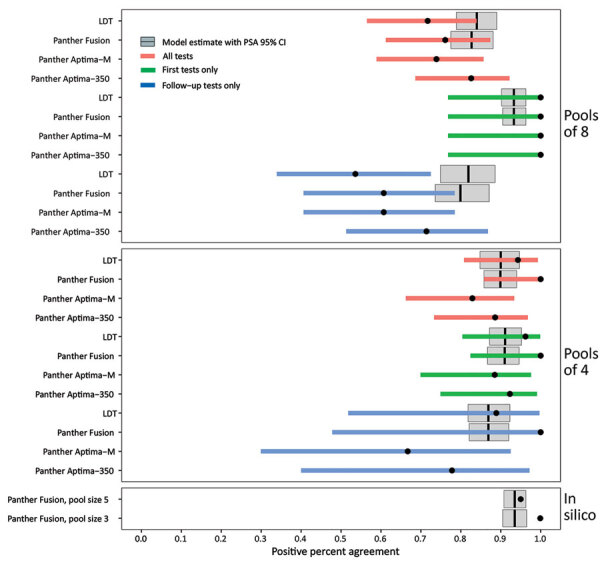
Performance of nucleic acid amplification tests for detection of severe acute respiratory syndrome coronavirus 2 in prospectively pooled specimens. Empiric and modeled estimates of positive percent agreement (PPA) with 95% CIs for each pool size, testing platform, and sample type (all versus first initial diagnostic versus follow-up). Black circles indicate empiric PPA point estimates, and colored horizontal bars indicate 95% CI. The 95% CI for the in silico data are too narrow to be visible in this plot. Gray boxplots indicate the modeled estimate of PPA, vertical black lines indicate the modeled PPA point estimate, and gray box indicates the 95% CI of the probabilistic sensitivity analysis. No modeled estimates are available for Panther Aptima because this is a transcription-mediated amplification assay, and the model is based on dilutional effects inherent to real-time PCR only. The empiric 95% CIs contain the modeled PPA point estimates for all conditions except for pools of 8 follow-up tests only and the in silico data. Data used to generate this figure are provided in Appendix Table 3. Panther Fusion and Panther Aptima are from Hologic (https://www.hologic.com). LDT, laboratory-developed test; PSA, probabilistic sensitivity analysis.

One-way deterministic and probabilistic sensitivity analyses incorporating uncertainty in the underlying model assumptions of dilutional effect and probit regression shape demonstrate a moderate (±2% to ±7%) effect on PPA, which is more pronounced with larger pool sizes and proportion of C_t_ values above the LoD (Appendix Figure 7). In contrast, these parameters have a much smaller effect on testing efficiency (Appendix Figure 8). The 95% CIs for the empirically determined and modeled PPAs overlapped for most of the evaluated empiric datasets, although these values overestimated PPA for the LDT follow-up tests only subset (Figure 5). For the in silico validation data, the modeled PPA was similar for pool sizes of 5 and 3, despite in silico data analysis predicting a higher PPA for pools of 3. Modeled testing efficiency was actually slightly higher for pools of 3 than pools of 5, which was probably caused by the high prevalence of 19.1% in this dataset (Appendix Table 3).

## Discussion

In this study, >1,600 samples were tested in pool sizes of 8 and 4 by using 3 different SARS-CoV-2 platforms, and pooled testing showed decreased PPA relative to individual samples. False-negative results occurred exclusively in pools containing samples with low estimated viral load (C_t_ >34). Overlapping CIS in PPA and NPA at each pool size suggest that the lower test performance is inherent to the pooling process itself, rather than the assay. Although Panther Fusion C_t_ values were on average higher than those of the LDT, the negative proportional bias suggests that at low estimated viral loads (C_t_ >36), the Panther Fusion outperformed the LDT. This finding might be caused by the different targets of amplification (envelope gene versus open reading frame 1ab) or PCR efficiency. These subtle differences between the 2 assays highlight the method-dependent nature of test performance, a variable that cannot be anticipated, and therefore is not explicitly accounted for in most statistical models of pooled testing. Thus, method comparison studies should be performed before large-scale implementation of any pooled testing strategies, especially those that use different platforms for the pooled and individual stages of testing.

The findings of our study contrast with those of a recent study, which concluded that pooling in groups of 8 did not compromise test performance (*5*). This finding might be explained by differences in patient population, higher proportion of positive pools and rRT-PCR result interpretation. Another recent study of artificially constructed pools reported no major decrease in sensitivity in pools of <32 samples (*3*). This finding is probably explained by the relatively low starting C_t_ values of individual positive samples in this study; none exceeded a C_t_ of 30. However, this study and other experimental studies have shown empirical increases in pooled C_t_ values directly proportional to dilution factor, a relationship that was also observed in our study (*3*,*4*,*9*).

These differences highlight the effect of viral load distribution and assay analytical sensitivity on pooled test performance, both of which should be taken into account when choosing pool size and diagnostic assay. Although samples with C_t_ values >33 have not been reported to produce cultivable virus in convalescent phase COVID-19 patients (*21*), >15% of first-time diagnostic specimens in our laboratory were detected at a C_t_ >35. A similar proportion of weakly positive samples that had high C_t_ values at a public health department virology laboratory in New York has been described (S.B. Griesemer, unpub. data). Assays with lower analytical sensitivity may miss specimens with late C_t_ values, for which the potential associated burden of onward transmission is currently unclear.

The stochastic model in this study demonstrated that expected PPA between pooled and individual rRT-PCRs was highly dependent on assay analytical sensitivity (represented by 95% LoD), viral load distribution of test-positive patients (represented by proportion C_t_ >LoD), pool size, and disease prevalence (represented by proportion of tests positive). The model outputs were not always intuitive; larger pool sizes were not always less sensitive or more efficient. With increased prevalence, larger pool sizes were more sensitive because they were more likely to contain >1 positive sample/pool. They were also less efficient because a larger proportion were positive and required deconvolution.

The model output was largely independent of the actual LoD and viral load-to-C_t_ value relationship of a given assay, making it generalizable across different rRT-PCRs. The only input parameters it requires are the proportion of positive test results and the proportion of samples with C_t_ >LoD, both of which should be readily available to any laboratories conducting clinical testing. Future studies on the sensitivity of pooled testing strategies should report these parameters.

Previous models of pooled testing strategies for SARS-CoV-2 have primarily examined the effect of pool size and prevalence on testing efficiency but have not addressed the expected decrement in assay sensitivity that accompanies a putative increase in efficiency (*6*,*22*). Those studies that have examined sensitivity did not explicitly model the effect of variable viral load distribution of test-positive patients, a parameter that can vary based on the underlying patient population (asymptomatic versus symptomatic and severe versus nonsevere), purpose of testing (diagnostic versus follow-up), and specimen type (*8*,*23*–*27*). In addition, previous modeling studies and in silico analyses have mostly used the C_t_ cutoff value of the assay, assuming 100% detection below the cutoff value, and 0% detection above it. In contrast, our model incorporates the probabilistic nature of detection at and above the LoD, which better approximates reality.

Our approach is limited by the generalizability of the probit regression shape and the equation estimating dilutional effect, as demonstrated by the variability seen on probabilistic and deterministic sensitivity analysis. Furthermore, the model assumes that the PCR is 100% efficient and that it is devoid of any proportional bias between individual and pooled tests. In addition, the model might underestimate PPA and efficiency of pooled testing if samples in each pool are not independent; placing samples with higher pretest probability in the same pool would decrease the total number of positive pools and increase the likelihood of detection. This feature could be leveraged by pooling specimens from persons in the same household or social distancing pod, such as coworkers on the same shift or students sharing a classroom. These factors, among others, might be the reasons for which the probabilistic sensitivity analysis CIs often did not contain the empiric point estimate in our validation data. These unaccounted-for factors might limit the ability of the model to provide a reliable point estimate.

The strengths of our study include its relatively large sample size, prospective rather than experimental construction of pools, and assessment of 2 different pool sizes. It also compared 3 different SARS-CoV-2 assays, 2 of which are commercially available on highly automated platforms suitable for large-scale testing. Our study was limited by its assessment of only a 2-stage pooling strategy. An additional limitation includes selection bias because the proportion of positive test results in the study specimens was higher because of the inclusion of follow-up samples from known COVID-19 patients enrolled in clinical research studies. Finally, test performance might vary depending on specimen collection medium, which we did not assess in this study (S.B. Griesemer, unpub. data).

In conclusion, a 2-stage pooled testing strategy for detection of SARS-CoV-2 by nucleic acid amplification is feasible and has the potential to strongly increase testing capacity. However, increased pool size and efficiency can compromise PPA. More studies examining early viral load kinetics and infectiousness are needed to fully evaluate the risks versus benefits of pooled testing. We provide a model to predict optimal pool size and associated expected PPA based on limit of detection, C_t_ value distribution, and proportion of positive test results. If this model can be externally validated, it might be useful in guiding SARS-CoV-2 pooled testing in other laboratories and as part of an adaptive risk-based strategy.

Appendix Additional information on performance of nucleic acid amplification tests for detection of severe acute respiratory syndrome coronavirus 2 in prospectively pooled specimens.
