# Supplementary material for: Performance of Nucleic Acid Amplification Tests for Detection of Severe Acute Respiratory Syndrome Coronavirus 2 in Prospectively Pooled Specimens
Source: Emerg Infect Dis. 2021 Jan;27(1):92–103. doi: 10.3201/eid2701.203379 (PMC7774575; doi:10.3201/eid2701.203379)
Supplement: Appendix — Additional information on performance of nucleic acid amplification tests for detection of severe acute respiratory syndrome coronavirus 2 in prospectively pooled specimens. [file 20-3379-Techapp-s1.pdf]

Article DOI: https://doi.org/10.3201/eid2701.203379

Performance of Nucleic Acid Amplification Tests for Detection of Severe Acute Respiratory Syndrome Coronavirus 2 in Prospectively Pooled Specimens

Appendix

Assay Comparisons for Pools of 4

To evaluate a pool size of 4, a total of 192 pools from 768 unique samples were tested on 3 different NAAT platforms (Table 1). Because of unforeseen logistical considerations, 56 of the 192 pools were tested only by laboratory-developed test (LDT) and Panther Aptima, but not tested by Panther Fusion. The remaining 136 pools were tested by all 3 methods. Among the 768 individual samples, 38 (4.9%) were positive, with a median cycle threshold (C_t) value of 29.3 (95% CI 20.3–33.9). First-time diagnostic samples had higher median C_t values than follow-up tests (Table 2).

Among the tested pools of 4, 18.2% (35/192) contained $\geq$1 positive sample. The positive pools were comprised of 32 pools with 1 positive sample, and 3 pools with 2 positive samples (Appendix Table 2). There were no false-positive pools. The overall positive percent agreement (PPA) of pooled testing ranged from 82.9% to 100% (Table 3). The 26 pools containing positive first-time diagnostic samples had higher PPA than the 9 pools containing positive follow-up tests by LDT (Appendix Table 3).

There were 6 total pools for which $\geq$1 method was falsely negative, all of which contained only 1 positive specimen. Each of these 6 specimens had an individual C_t value >34 cycles (median 36.4, interquartile range 34.6–37.5). Among individual positive specimens in the pools of 4 dataset, 10 (26.3%) had a C_t>34. For the LDT, Panther Fusion, Panther Aptima-M, and Panther Aptima-350, 2/10 (20.0%), 0/6 (0.0%, 4 samples were not subjected to pooled testing), 6/10 (60.0%), and 4/10 (40.0%), respectively, were false negative. Four samples were first-time diagnostic specimens from persons who were either symptomatic or had suspected exposures; the other 2 were follow-up tests in persons with a previous diagnosis of COVID-19.

Linearity Studies for Pools of 4

For pools containing only 1 positive sample, the pooled Panther Fusion assay showed positive systematic bias when compared with the individual LDT assay, as shown by the Passing-Bablok regression intercept value being >0. By LDT, pools resulted an average of 2.2 cycles (95% limits of agreement 0.6–3.9; $p<0.001$) later than the individual positive samples (Appendix Figure 3, panels A, B). By Panther Fusion, pools resulted an average of 3.1 cycles (0.8–5.3; $p<0.001$) later than the individual positive samples (Appendix Figure 3, panels C, D). Pools resulted an average of 0.73 cycles (−1.06 to 2.53; $p<0.001$) later on Panther Fusion when compared with the LDT (Appendix Figure 3, panels E, F). There was minimal proportional bias among the 3 assays, although the 95% CIs for the Passing-Bablok regression slope for individual LDT versus pooled LDT and for pooled LDT versus pooled Panther Fusion do not contain 1. This finding indicates slight positive and negative proportional biases, respectively. The proportional bias is additionally highlighted in the Bland-Altman plots, which demonstrate that at higher C_t values, Panther Fusion outperforms the LDT.

External In Silico Validation Data

Data from an in silico sensitivity analysis for the Panther Fusion assay was obtained (Hologic Inc.) to validate our model. The data includes 52,272 tests for severe acute respiratory syndrome coronavirus 2 (SARS-CoV-2) performed during March–July 2020 at 2 external sites with an average prevalence of 19.1% (Table 2), and 13.0% of positive specimens with a C_t above that corresponding to the limit of detection of 35.6. The in silico sensitivity analysis was performed according to US Food and Drug Administration recommendations by first determining the expected shift in C_t values using the Passing-Bablok regression equation generated through verification testing (*1*). The expected PPA was calculated by dividing the number of specimens with a shifted C_t value beyond the cutoff of the assay by the total number of specimens tested. Pool sizes of 5 and 3 were evaluated in this manner, with expected PPA of 95.0% (94.7–95.2) and 99.9% (99.9–99.9), respectively (Appendix Table 3).

Modeling

We developed a stochastic simulation model to estimate PPA and efficiency for a 2-stage pooled testing algorithm, which was based on characteristics of the underlying assay and patient population. To study the effect on PPA and efficiency, we varied the proportion of positive tests (*s:* 0.1%, 1.0%, 3.0%, 5.0%, 10.0%, 15.0%), the 95% assay limit of detection C_t value (LoD; *l:* C_t corresponding to 95% detection: 32–40), the percentage of individual amplified C_t values above the LoD (*x:* 5.0%, 10.0%, 15.0%, 20.0%, 25.0%, 30.0%), and pool size (*p:* 1–20).

We fit the C_t values of samples positive for SARS-CoV-2 by real-time reverse transcription PCR (n = 804) received from an independent set of unique patients undergoing testing for SARS-CoV-2 during March 1–June 24, 2020 (n = 66,070) to candidate continuous probability distributions, and selected the best-fitting distribution based on the Bayesian information criterion and Kolmogorov-Smirnov statistic (0.0436). Because of slight negative skewness, a Weibull distribution best fit these data with shape and scale parameters of 4.55 and 29.86 (Appendix Figure 4). We then generated a set of random C_t values by sampling 50,000 times from this distribution. To study differing scenarios in which a variable proportion of samples had viral loads below the LoD, we generated additional sets of 50,000 C_t values with 5%–30% of values above each LoD. For the base case, the expected C_t value of the pool was calculated by using the following equation: $C_{tpoolexpected} = –\log_2((\sum 2^{-Ctsingle})/poolsize)$. To model the probabilistic nature of detecting RNA at a given calculated pooled C_t value, we first fit a probit regression model by using binary detection from an independent LoD experiment (100, 200, 500, 1,000, 2,000, 2,500, 5,000, and 10,000 copies/mL in replicates of 5–20). This experiment's LoD and confidence interval (685 copies/mL [95% CI 484–1,074], C_t 35.9 [35.3–36.5]) were incorporated into the base case and sensitivity analyses.

For each pool size *p*, prevalence *s*, LoD *l*, and proportion of randomly-generated C_t values above LoD *x*, we randomly generated 10,000 pools for each possible combination of negative and positive pools (1 + *p*). Pools with zero positive samples were considered to be true negatives. For each randomly-generated pool with positive samples, we calculated the expected pooled C_t value from the individual randomly-sampled C_t values and assigned each pool as a true-positive result or a false-negative result based upon the probability of detection derived from the probit regression model at a given LoD and expected pooled C_t value. Estimated PPA (true positives/[true positives + false negatives]) and average tests expected per sample ([1 + *p**(true positive pools)]/*p*) were calculated, and results were weighted by the probability of observing a given pool combination with *i* individual positive samples (from 1 to *p*) by using a binomial distribution. Negative percent agreement was assumed to be 100%. Because the input datasets used to train this model were independent from the pooled datasets, we subsequently validated modeled estimates of PPA and average tests expected per sample against our empirical data for pools of 8 and 4, as well as external in silico data for pools of 5 and 3. Beyond the external factors that contributed to model estimates (individual C_t values, prevalence, proportion of samples above LoD), we also assessed model (robustness by using deterministic and probabilistic sensitivity analyses by varying pooling dilution $C_{tpoolexpected} = –\log_2((\sum 2^{-Ctsingle})/poolsize \pm 1\ C_t$) and sampling from the 95% CI of the probit regression. The implementation

code and data used to generate and validate this model is available upon request to the corresponding author.

Reference

1. Food and Drug Administration. Molecular Diagnostic Template for Laboratories, July 28, 2020 [cited 2020 Sep 18]. https://www.fda.gov/media/135658/download

Appendix Table 1. Receiver operating characteristic curve table for Panther Aptima, based on pools of 8 containing a single positive sample (n = 36)*

Positive percent agreement	Negative percent agreement	Relative light unit threshold
1.000	0.047	301
0.972	0.109	302
0.972	0.156	304
0.972	0.188	305
0.972	0.203	306
0.972	0.219	307
0.972	0.234	308
0.944	0.297	309
0.944	0.359	310
0.944	0.406	311
0.944	0.438	312
0.944	0.469	313
0.944	0.500	314
0.944	0.516	315
0.917	0.578	316
0.889	0.641	317
0.861	0.688	318
0.833	0.688	320
0.833	0.703	321
0.833	0.750	322
0.833	0.797	323
0.833	0.844	325
0.833	0.891	326
0.833	0.906	327
0.833	0.922	328
0.806	0.938	330
0.778	0.953	332
0.778	0.984	343
0.750	0.984	393
0.750	1.000	474
0.722	1.000	531
0.694	1.000	570

*Cases defined as pools containing ≥1 positive samples by individual testing; controls defined as pools containing only samples negative by individual testing. Ten pools containing >1 positive sample were excluded from analysis.

Appendix Table 2. Results of 4-sample pooled testing, by testing platform and number of positive specimens per pool (n = 192)*

	Pooled testing				Individual testing		
Characteristic	LDT	Panther Fusion	Panther Aptima-M	Panther Aptima-350	Positive (no. 1 PP, no. >1 PP)	Negative	Total no. pools
	+	+	+	+	22 (19, 3)	0	22
	+	+	–	+	1 (1, 0)	0	1
	+	+	–	–	1 (1, 0)	0	1
	+	NA	+	+	7 (7, 0)	0	7
	+	NA	–	+	0 (0, 0)	0	0
	+	NA	–	–	2 (2, 0)	0	2
	+	–	+	+	0 (0, 0)	0	0
	+	–	–	+	0 (0, 0)	0	0
	+	–	–	–	0 (0, 0)	0	0
	–	+	+	+	0 (0, 0)	0	0
	–	+	–	+	0 (0, 0)	0	0
	–	+	–	–	0 (0, 0)	0	0
	–	NA	+	+	0 (0, 0)	0	0
	–	NA	–	+	1 (1, 0)	0	1
	–	NA	–	+	1 (1, 0)	45	46
	–	–	+	+	0 (0, 0)	0	0
	–	–	–	+	0 (0, 0)	0	0
	–	–	–	–	0 (0, 0)	112	112
No. positive pools	33	24	29	31	35 (32, 3)	–	–
No. negative pools	159	112	163	161	–	157	–
Total no. pools	192	136	192	192	–	–	192

*LDT, laboratory-developed test; NA, not applicable, indicates pools with no Panther Fusion testing done; Panther Aptima-M, Panther Aptima with manufacturer-set relative light unit cutoff value; Panther Aptima-350, Panther Aptima with relative light unit cutoff value >350 was considered positive; C_t, cycle threshold; 1 PP, 1 positive specimen in pool; ≥1 PP, ≥2 positive specimens in pool; RLU, relative light unit; –, negative; +, positive..

Appendix Table 3. Modeled versus empiric positive percent agreement and testing efficiency*

Testing platform	Pool size	Test type	Model input variables: Positive samples, %	Model input variables: 95% LoD	Model input variables: C_t >LoD, %	Model estimate with PSA: PPA, % (95% CI)	Model estimate with PSA: Tests/sample (95% CI)	Empiric data: PPA, % (95% CI)	Empiric data: Tests/sample
LDT	8	All	6.6	35.9	22.4	84.0 (78.9–89.0)	0.479 (0.457–0.500)	71.7 (56.5–84.0)	0.434
Panther Fusion	8	All	6.6	35.6	24.1	82.7 (77.5–88.1)	0.473 (0.451–0.496)	76.1 (61.2–87.4)	0.452
Panther Aptima-M	8	All	–	–	–	–	–	73.9 (58.9–85.7)	0.434
Panther Aptima-350	8	All	–	–	–	–	–	82.6 (68.6–92.2)	0.470
LDT	8	First†	3.7	35.9	8.3	93.3 (90.2–96.3)	0.368 (0.360–0.376)	100 (76.8–100.0)	–
Panther Fusion	8	First†	3.7	35.6	8.3	93.3 (90.5–96.3)	0.368 (0.361–0.376)	100 (76.8–100.0)	–
Panther Aptima-M	8	First†	–	–	–	–	–	100 (76.8–100.0)	–
Panther Aptima-350	8	First†	–	–	–	–	–	100 (76.8–100.0)	–
LDT	8	Follow-up†	15.2	35.9	32.4	81.9 (74.9–88.6)	0.725 (0.680–0.774)	53.6 (33.9–72.5)	–
Panther Fusion	8	Follow-up†	15.2	35.6	35.3	79.9 (73.6–87.1)	0.711 (0.664–0.763)	60.7 (40.6–78.5)	–
Panther Aptima-M	8	Follow-up†	–	–	–	–	–	60.7 (40.6–78.5)	–
Panther Aptima-350	8	Follow-up†	–	–	–	–	–	71.4 (51.3–86.8)	–
LDT	4	All	4.9	35.9	15.8	90.0 (84.8–94.7)	0.414 (0.406–0.422)	94.3 (80.8–99.3)	0.422
Panther Fusion‡	4	All	4.9	35.6	15.8	89.9 (85.8–94.0)	0.414 (0.406–0.421)	100 (85.8–100)	0.426
Panther Aptima-M	4	All	–	–	–	–	–	82.9 (66.2–93.4)	0.401
Panther Aptima-350	4	All	–	–	–	–	–	88.6 (73.3–96.8)	0.411
LDT	4	First§	5.7	35.9	14.3	91.1 (87.2–95.2)	0.441 (0.432–0.449)	96.2 (80.4–99.9)	–
Panther Fusion‡	4	First§	5.7	35.6	14.3	91.0 (86.7–94.6)	0.440 (0.431–0.448)	100 (82–100.0)	–
Panther Aptima-M	4	First§	–	–	–	–	–	88.5 (69.9–97.6)	–
Panther Aptima-350	4	First§	–	–	–	–	–	92.3 (74.9–99.1)	–
LDT	4	Follow-up§	3.6	35.9	20.0	86.9 (81.8–92.3)	0.369 (0.362–0.376)	88.9 (51.8–99.7)	–
Panther Fusion‡	4	Follow-up§	3.6	35.6	20.0	86.9 (82.1–92.0)	0.369 (0.362–0.376)	100 (47.8–100.0)	–
Panther Aptima-M	4	Follow-up§	–	–	–	–	–	66.7 (29.9–92.5)	–
Panther Aptima-350	4	Follow-up§	–	–	–	–	–	77.8 (40.0–97.2)	–
Panther Fusion	5	In silico	19.1	35.6	13.0	93.5 (90.8–96.3)	0.811 (0.793–0.830)	95.0 (94.7–95.2)	–
Panther Fusion	3	In silico	19.1	35.6	13.0	93.5 (90.5–96.5)	0.773 (0.759–0.787)	99.9 (99.9–99.9)	–

*Panther Aptima-M, Panther Aptima with manufacturer-set relative light unit cutoff; Panther Aptima-350, Panther Aptima with relative light unit cutoff value >350 was considered positive. C_t, cycle threshold; LDT, laboratory-developed test; LoD, limit of detection; PPA, positive percent agreement; PSA, probabilistic sensitivity analysis; –, negative.

†For a pool size of 8, there were 14 pools containing positive first test results only, and 28 pools containing positive follow-up test results only. These numbers represent the denominator for the calculation of PPA.

‡A total of 56 of the 192 pools tested on the other platforms were not tested by Panther Fusion.

§For a pool size of 4, there were 26 pools containing positive first test results only, and 9 pools containing positive follow-up test results only. These numbers represent the denominator for the calculation of PPA.

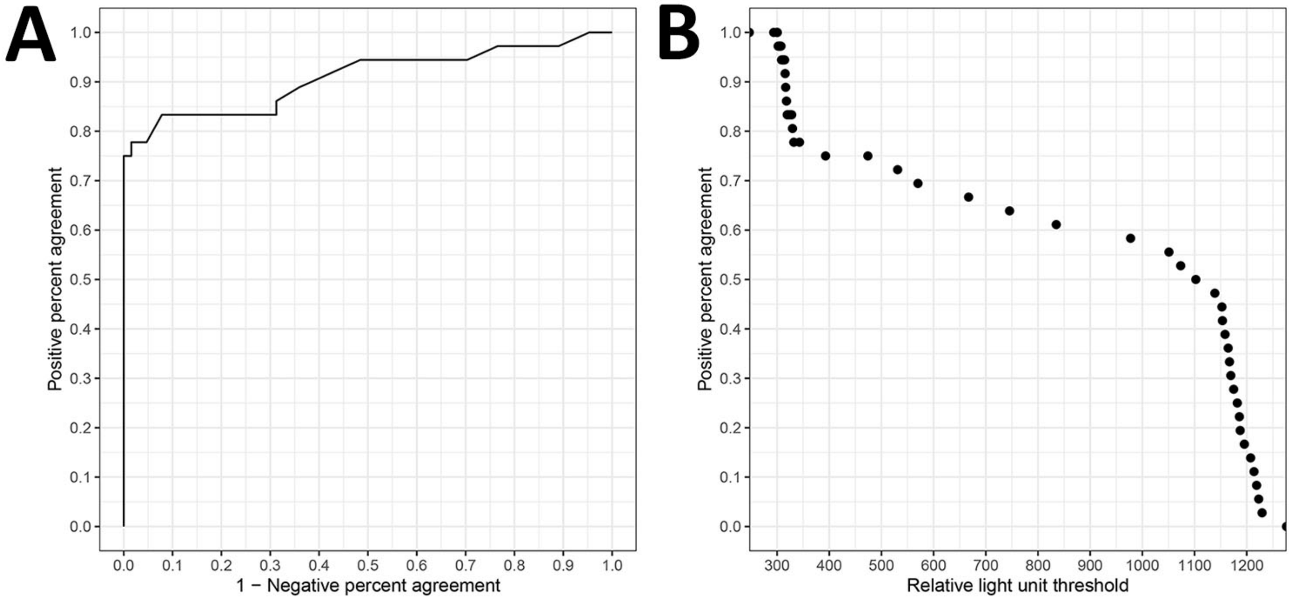

Appendix Figure 1. A) Receiver operating characteristic curve of pools of 8 containing only a single positive sample tested by Panther Aptima, and individual samples tested by LDT, with area under the curve of 0.911 (n = 36). B) Positive percent agreement (PPA) plotted against Panther Aptima relative light unit (RLU) threshold. Based on the inflection point of this curve, an RLU cutoff of >350 was chosen to maximize PPA.

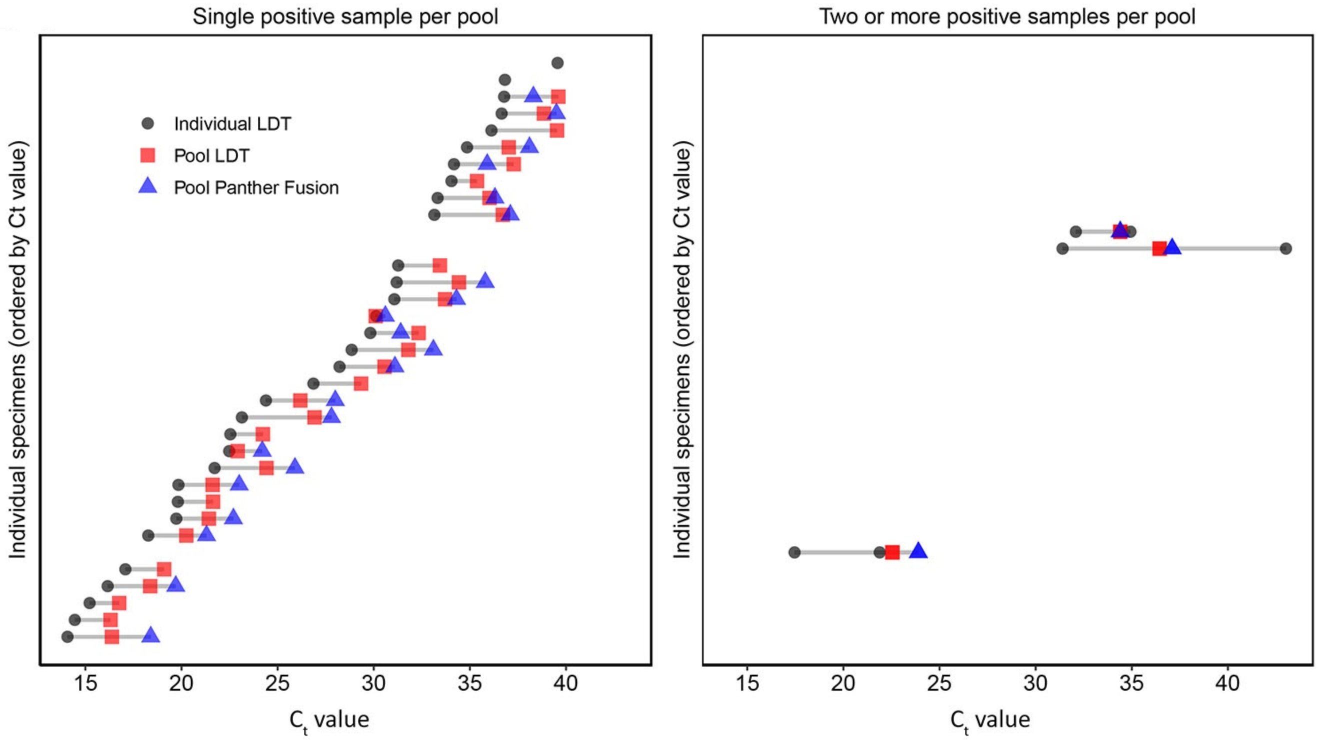

Appendix Figure 2. For a pool size of 4, paired individual and pooled C_t values for each individually positive sample (n = 38), in order of increasing individual C_t value. The left panel contains pools comprised of only a single positive sample. The right panel contains pools comprised of two or more positive samples. The gray lines span the range of C_t values associated with a given pool. Pools without a red square were false negatives by the laboratory-developed test (LDT). Pools without a blue triangle were not tested by Panther Fusion, and do not represent false negatives.

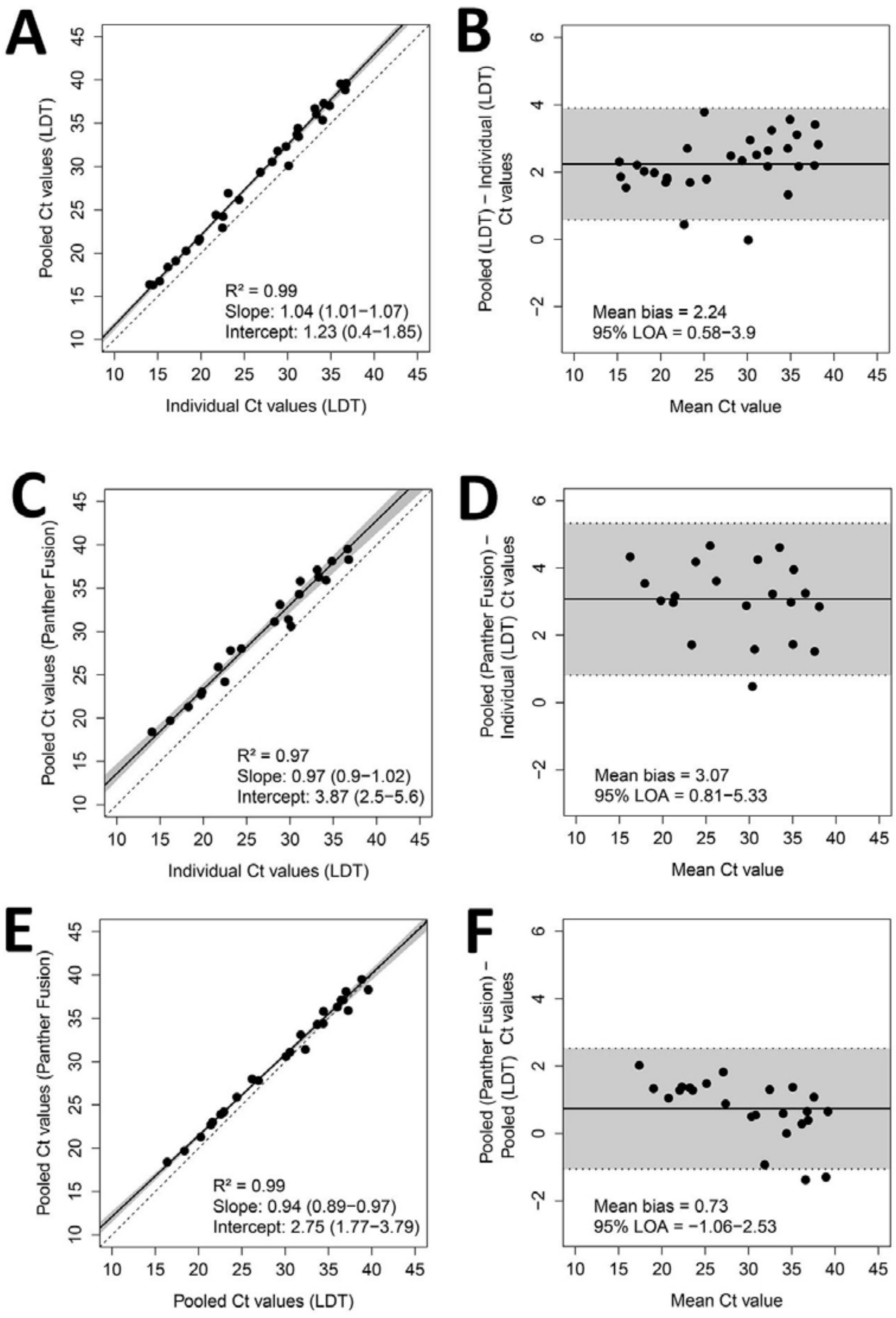

Appendix Figure 3. Passing-Bablok regression and Bland-Altman plots for pools of 4 containing only a single positive sample, tested by A and B) pooled LDT versus individual LDT (n = 30), C and D) pooled Panther Fusion versus individual LDT (n = 21), and E and F) pooled Panther Fusion versus pooled LDT (n = 24). For the Passing-Bablok regression plots (A, C, E), the solid line represents the line of regression, with 95% confidence interval shaded in gray. The dashed line represents the line of identity. The slope and intercept of the regression line are reported with 95% confidence intervals in parentheses. For the Bland-Altman plots (B, D, F), the solid line represents the mean difference in Ct value, with 95% limits of agreement range shaded in gray.

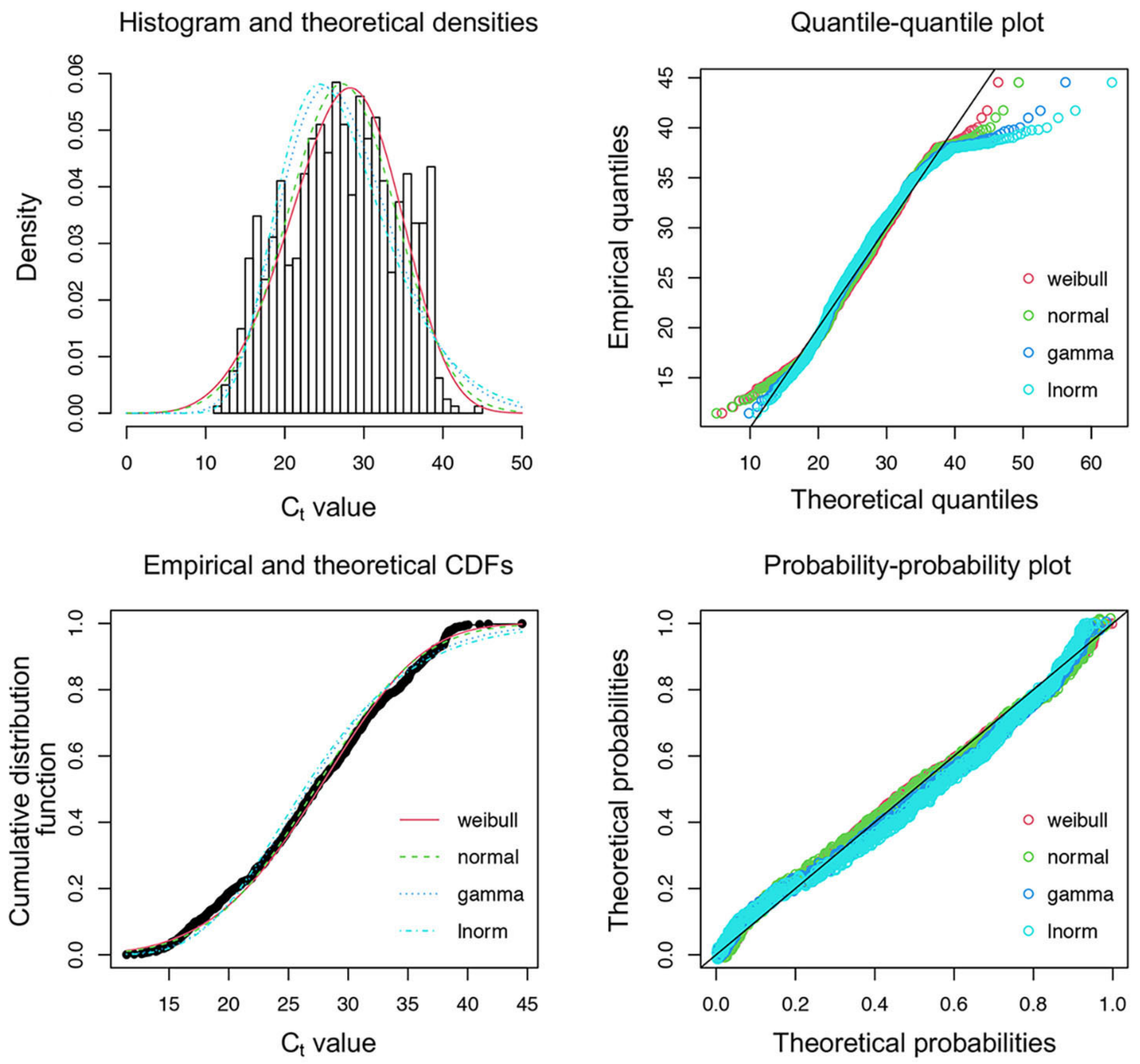

Appendix Figure 4. Continuous probability distributions fit to independent dataset of cycle threshold (C_t) values not subjected to pooled testing. Fitted theoretical weibull (red), normal (green), gamma (dark blue), and log-normal (light blue) distributions are plotted alongside empirical dataset for probability densities, quantiles (Q-Q plot), cumulative distribution functions, and probabilities (P-P plot). The fitted Weibull distribution was selected on the basis of minimization of the Bayesian Information Criterion, Akaike information criterion, and Kolmogorov–Smirnov statistic.

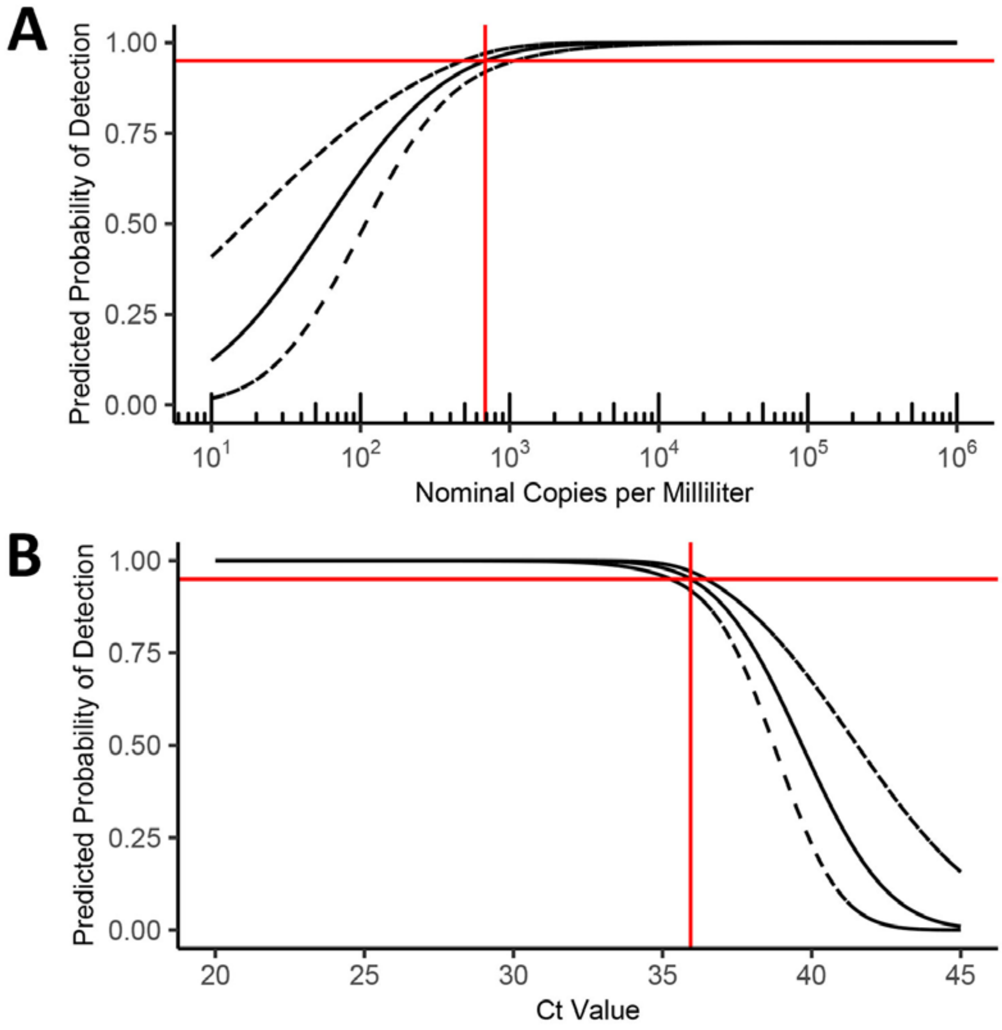

Appendix Figure 5. Fitted probit regression (solid black line) with 95% confidence intervals (dashed black lines) derived from independent limit of detection (LoD) experiment. Probability of detection is plotted against nominal viral copies per milliliter (A, top) and corresponding cycle threshold (C_t) value (B, bottom). Solid red lines indicate 95% estimated probability of detection (horizontal) and corresponding 95% LoD (vertical, 685 cp/mL or C_t 35.9).

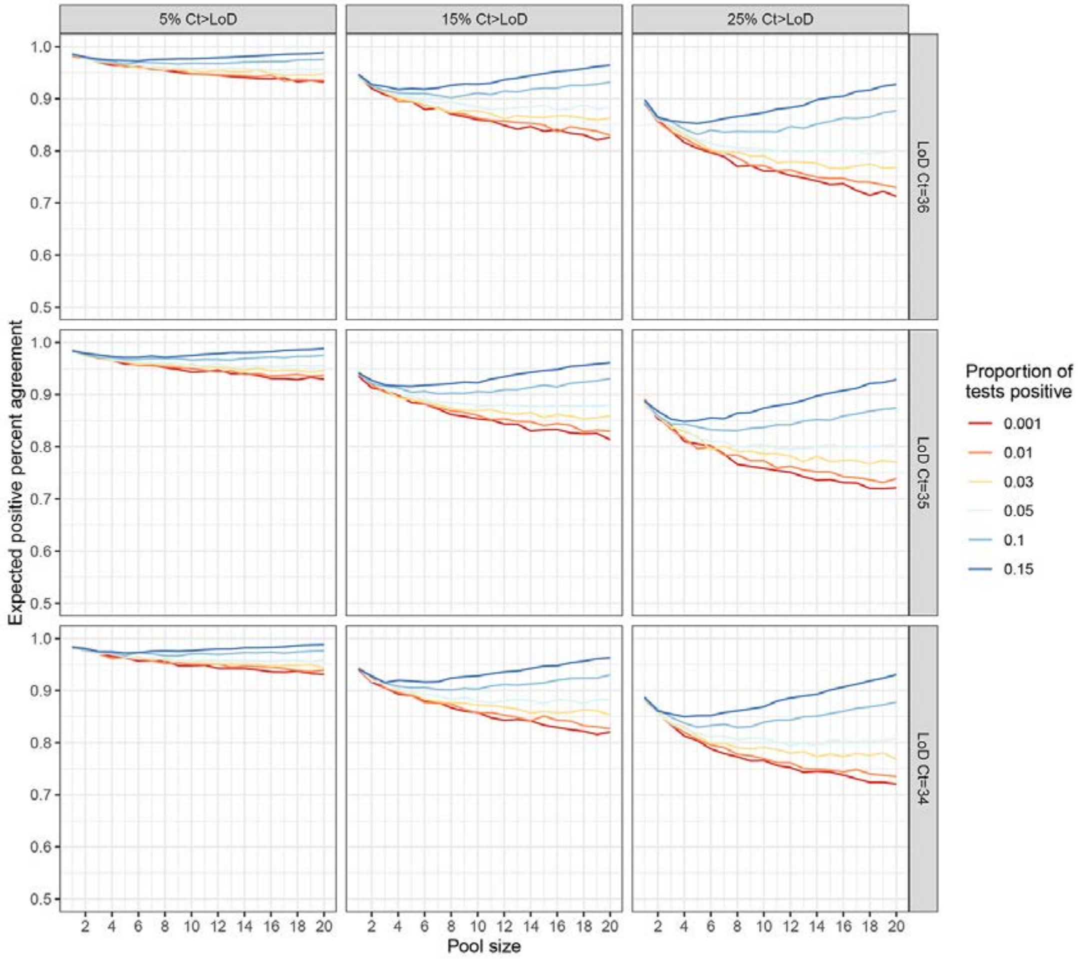

Appendix Figure 6. Model-estimated positive percent agreement (PPA) and testing efficiency, by pool size, proportion of tests positive, assay sensitivity represented by cycle threshold (C_t) corresponding to the 95% limit of detection (LoD), and proportion of samples with C_t above the LoD. The relationship between PPA and pool size is independent of the actual C_t value corresponding to the 95% LoD due to a fixed proportion of C_t values above the LoD (5%, 15%, 25%), demonstrated by identical plots in each vertical panel.

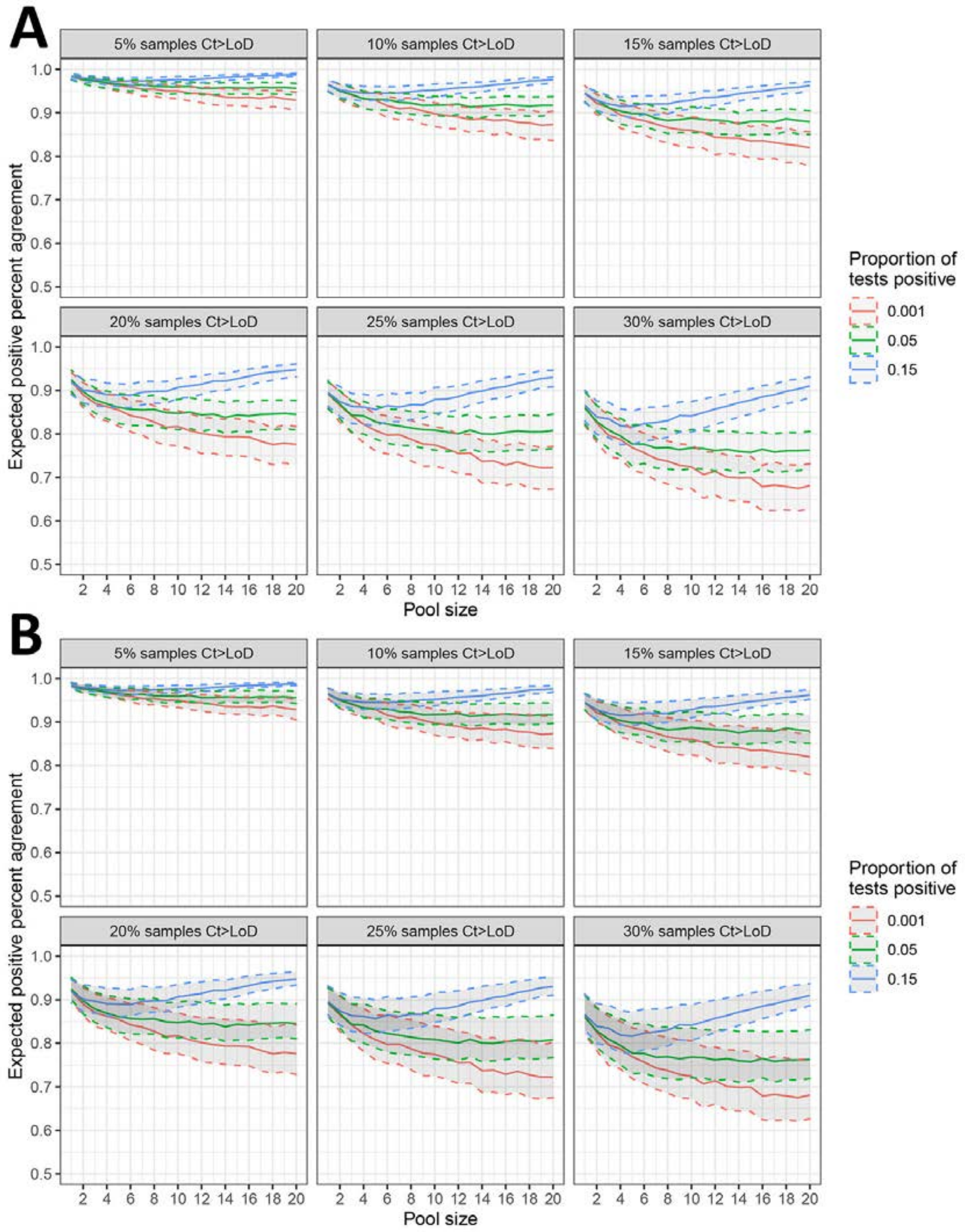

Appendix Figure 7. One-way deterministic sensitivity analysis for modeled estimates of positive percent agreement (PPA) between pooled and individual testing at pool sizes from 1–20 for variable prevalence. Solid lines indicate modeled base case estimates (Figure 4, panel A), while dashed lines indicate modeled estimates at upper and lower bounds of sensitivity analysis. A) Deterministic sensitivity analysis for deviation from pooled testing dilution effect (±1 C_t value). B) Deterministic sensitivity analysis for deviation from fitted probit regression (±2 SDs).

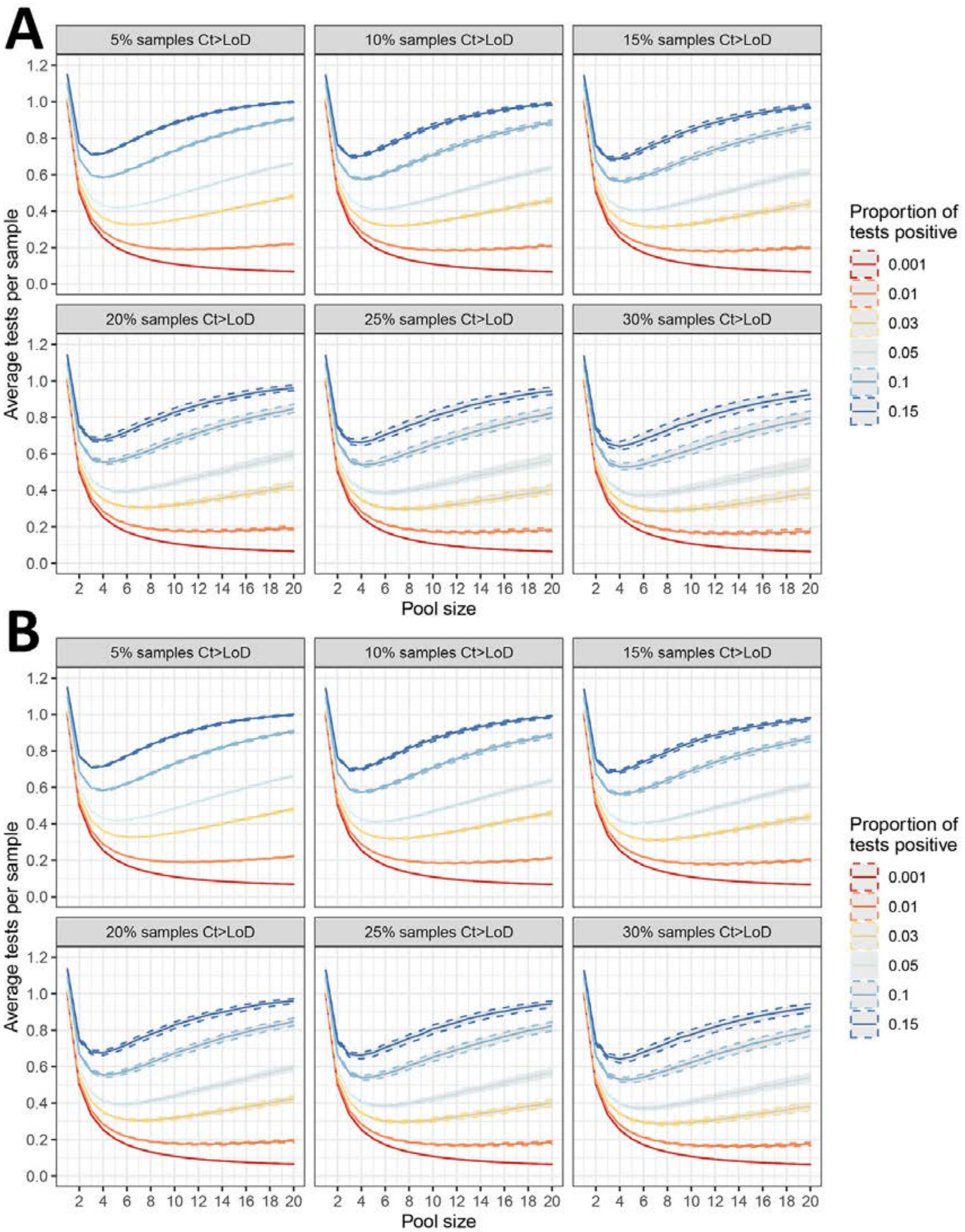

Appendix Figure 8. One-way deterministic sensitivity analysis for modeled estimates of tests per sample between pooled and individual testing at pool sizes from 1–20 for variable prevalence. Solid lines indicate modeled base case estimates (Figure 4, panel B), while dashed lines indicate modeled estimates at upper and lower bounds of sensitivity analysis. A) Deterministic sensitivity analysis for deviation from pooled testing dilution effect (±1 C_t value). B) Deterministic sensitivity analysis for deviation from fitted probit regression (±2 SDs).
